# Oxidative stress, mitochondrial dysfunction and calcium overload in human lamina cribrosa cells from glaucoma donors

**Published:** 2011-05-05

**Authors:** E.M. McElnea, B. Quill, N.G. Docherty, M. Irnaten, W.F. Siah, A.F. Clark, C.J. O’Brien, D.M. Wallace

**Affiliations:** 1 Institute of Ophthalmology, Mater Misericordiae University Hospital, Dublin, Ireland; 2 University College Dublin School of Medicine-Medical Science, University College Dublin, Dublin, Ireland; 3 Department of Cell Biology & Anatomy and the North Texas Eye Research Institute, University North Texas Health Science Center, Ft. Worth, TX

## Abstract

**Purpose:**

Oxidative stress is implicit in the pathological changes associated with glaucoma. The purpose of this study was to compare levels of oxidative stress in glial fibrillary acid-negative protein (GFAP) lamina cribrosa (LC) cells obtained from the optic nerve head (ONH) region of 5 normal (NLC) and 4 glaucomatous (GLC) human donor eyes and to also examine mitochondrial function and calcium homeostasis in this region of the ONH.

**Methods:**

Intracellular reactive oxygen species (ROS) production was examined by a thiobarbituric acid reactive substances (TBARS) assay which measures malondialdehyde (MDA), a naturally occurring product of lipid peroxidation and is used as an indicator of oxidative stress. Mitochondrial membrane potential (MMP) and intracellular calcium ([Ca^2+^]_i_) levels were evaluated by flow cytometry using the JC-1 (5,5′,6,6′-tetrachloro-1,1′,3,3′-tetrabenzimidazolecarbocyanine iodide) and fluo-4/AM probes respectively. Anti-oxidant and Ca^2+^ transport system gene and protein expression were determined by real time polymerase chain reaction (RT-PCR) using gene-specific primer/probe sets and western immunoblotting, respectively.

**Results:**

Intracellular ROS production was increased in GLC compared to NLC (27.19±7.05 µM MDA versus 14.59±0.82 µM MDA, p<0.05). Expression of the anti-oxidants Aldo-keto reductase family 1 member C1 (AKR1C1) and Glutamate cysteine ligase catalytic subunit (GCLC) were significantly lower in GLC (p=0.02) compared to NLC control. MMP was lower in GLC (57.5±6.8%) compared to NLC (41.8±5.3%). [Ca^2+^]_i_ levels were found to be higher (p<0.001) in GLC cells compared to NLC. Expression of the plasma membrane Ca^2+^/ATPase (PMCA) and the sodium-calcium (NCX) exchangers were lower, while intracellular sarco-endoplasmic reticulum Ca^2+^/ATPase 3 (SERCA) expression was significantly higher in GLC compared to NLC. Subjection of NLC cells to oxidative stress (200 µM H_2_0_2_) reduced expression of Na^+^/Ca2^+^  exchanger 1 (*NCX 1*), plasma membrane Ca2+ ATPase 1 (*PMCA 1*), and *PMCA 4* as determined by RT–PCR.

**Conclusions:**

Our data finds evidence of oxidative stress, mitochondrial dysfunction and impaired calcium extrusion in GLC cells compared to NLC cells and suggests their importance in the pathological changes occurring at the ONH in glaucoma. Future therapies may target reducing oxidative stress and / or [Ca^2+^]_i_.

## Introduction

Glaucoma is a neurodegenerative disease of the eye that is one of the leading causes of visual impairment and blindness worldwide [1,2]. It is a heterogeneous group of conditions that share a similar final common pathway of retinal ganglion cell (RGC) loss resulting in characteristic visual field loss. The lamina cribrosa (LC) is located within the optic nerve head (ONH) region and provides structural support for the RGC axons exiting the eye to form the optic nerve. There is loss of axons, excavation of the ONH and collapse of the LC in glaucoma [3,4]. Our group has shown that glial fibrillary acid-negative protein (GFAP) negative LC cells contribute to extracellular matrix (ECM) remodeling of the ONH in glaucoma [5-7]. A variety of glaucoma related stimuli such as transforming growth factor beta (TGF-β; a profibrotic mediator elevated in the glaucomatous ONH), cyclic mechanical stretch (increased intraocular pressure) and hypoxia (ONH ischemia) each increased the expression of ECM genes associated with glaucomatous ONH remodeling. These biomechanical and structural changes produce optic disc cupping and may prevent anterograde and retrograde axoplasmic flow at the LC (the mechanical theory of glaucoma) and/or reduce the perfusion pressure in the blood vessels of this region (the vascular theory of glaucoma). Oxidative stress is intricately associated with ischemic injury and therefore is likely to play a significant role in the pathogenesis of glaucoma.

Oxidative stress is defined as an increase over physiologic values of the intracellular concentration of reactive oxygen species (ROS). These ROS are free radicals containing one or more unpaired electrons which can damage a wide variety of biomolecules and cell structures. An imbalance between pro-oxidative and anti-oxidant capacity has been postulated to be a crucial feature in early retinal injury and glaucoma pathology [8,9] as well as being implicated in several animal studies involving elevated intraocular pressure [10-12].

Mitochondria are the most important endogenous source of ROS. Oxidative phosphorylation in these organelles results in electron leak that provides continuous formation of ROS which can directly damage the mitochondrion as well as other intracellular structures. Aberrant Ca^2+^ homeostasis, mitochondrial dysfunction and oxidative cell injury are known to be associated with a variety of neurodegenerative diseases, including glaucoma [13-22]. In addition, defects in the function of mitochondria have been shown to promote Ca^2+^ stress in glaucomatous trabecular meshwork (TM) cells [23]. The consequent mishandling of intracellular calcium by glaucomatous TM cells may contribute to the failure of this tissue leading to increased aqueous humor outflow resistance and elevated intraocular pressure.

Ca^2+^ is a ubiquitous intracellular messenger that is essential to the normal functioning of cells [24]. It plays a dual role as a second messenger as well as a stressor for cell damage and cell death/survival. Disturbances in Ca^2+^ homeostasis have been implicated in a diverse range of pathological conditions [25]. Our laboratory has previously studied mechano-sensitive pathways in the normal lamina cribrosa (NLC) cell and shown that cell membrane stretch induces intracellular Ca^2+^ signaling [26]. At any moment in time, intracellular Ca^2+^ homeostasis is determined by a balance between the channels that introduce Ca^2+^ into the cytoplasm and absorbers or extruders through which this signal is removed from the cytoplasm by the combined action of buffers, pumps and exchangers [27,28].

Key components in regulating Ca^2+^ homeostasis in cells include the Na^+^/Ca2^+^  exchanger (NCX) and plasma membrane Ca2+ ATPase (PMCA) channels which actively remove Ca^2+^ from the cells, and sarco/endoplasmic reticulum Ca2+ ATPase (SERCA) that sequesters Ca^2+^ in the endoplasmic reticulum (ER) [27-29]. All cells use these components as tools with which to maintain Ca^2+^ homeostasis. In pathologic conditions, excessive entry of Ca^2+^ into the mitochondria triggers calcium release from mitochondria [18], which can in turn induce further Ca^2+^ release via mitochondrial Ca^2+^-induced Ca^2+^ release [16,17]. Commonly, intracellular Ca^2+^ elevation is multi-phasic and includes Ca^2+^ release from additional intracellular stores and Ca^2+^ influx across the plasma membrane [30,31]. Interestingly, ROS can damage these plasma membrane proteins which are responsible for sustaining Ca^2+^ concentration gradients [32,33] and oxidative stress can also affect expression and/or activity of the aforementioned proteins [34,35].

We hypothesize that conditions of oxidative stress, mitochondrial dysfunction and dysregulation of calcium homeostasis could contribute to the pathological changes seen in the LC of glaucoma patients, in particular the glaucomatous remodeling of the ONH. Potential therapies could include the reduction of oxidative stress, dysfunctional mitochondria and Ca^2+^ signaling in LC cell.

## Methods

### Lamina cribrosa cell culture

Human GFAP-negative LC cells were obtained from donors with no history of glaucoma (NLC, n=5, mean age 77.8±6.38 years) and from donors with glaucoma (GLC, n=4, mean age 81.0±10.23 years; Alcon Research, Ltd, Fort Worth, TX). These cells are representative of a homogenous group of patients, are the healthy and diseased versions of the same cell type and have been previously characterized by the above laboratory [36]. Cultures were maintained in Dulbecco’s modified Eagle’s medium supplemented with 10% (v/v) fetal calf serum, 200m M L-glutamine, 10,000 units/ml penicillin and 10 mg/ml streptomycin (All reagents purchased from Sigma Aldrich, Wicklow, Ireland). Cultures were used in experimental procedures between passages 4 and 8.

To induce oxidative stress, post-confluent LC cultures were exposed to H_2_0_2_ (200 µM) for 1 h before analysis by real time polymerase chain reaction (RT-PCR) of expression levels of calcium exchangers/pumps (Na^+^/Ca2^+^  exchanger 1 [*NCX 1*], plasma membrane Ca2+ ATPase 1 [*PMCA 1*], and *PMCA 4*).

### Quantification of intracellular ROS production

Intracellular ROS production was quantified using the thiobarbituric acid reactive substance (TBARS) assay kit (Cell Biolabs Inc., San Diego, CA) Briefly, 1×10^6^ cells were collected in 1 ml of culture medium and sonicated for 3×5 s intervals at 40V over ice. SDS solution (100 µl) was added to the sample solution in a 5 ml vial and swirled to mix. The color reagent (4 ml) was then added and the vials boiled for 1 h before incubating on ice for 10 min. Vials were then centrifuged for 10 min at 1,600x g at 4 °C. Each sample (150 µl) was loaded (in duplicate) to a clear 96-well plate and the absorbance at 530–540 nm recorded. The content of malondialdehyde (MDA), a naturally occurring product of lipid peroxidation used as an indicator of oxidative stress was calculated for each sample form a standard curve.

### Mitochondrial membrane potential

The integrity of the mitochondrial membrane (ψm) was evaluated using the cationic dye JC-1. Briefly, 1×10^6^ cells were trypsinized and incubated at 37 °C for 30 min in 1 ml medium containing 2 µM JC-1 (Invitrogen, Dublin, Ireland). The cells were washed with PBS and the mean green fluorescence (FL-1 channel) and mean orange-red fluorescence (FL-2 channel) quantified using flow cytometry (BD FACSAria Cell Sorter; BD Biosciences, Dublin, Ireland). Ψm was expressed as the mean red to mean green fluorescence ratio. Non-stained control cells were included to evaluate baseline fluorescence.

### Measurement of intracellular calcium by flow cytometry

For flow cytometric analyses, the cells were cultured in T75 culture flasks at a density of 2×10^6^ cells and loaded with 2 µM fluo-4/AM (Bio-Sciences, Dublin, Ireland) for 45 min. The cells were then trypsinized, washed twice with cold isotonic solution, re-suspended in 1 ml isotonic solution (The osmolarity of the isotonic solution was 323±6 mOsm and consisted of [mM]: 120 NaCl; 6 KCl; 1 MgCl_2_; 2 CaCl_2_ 5.4 HEPES, and 80 mM D-Mannitol, [osmolarity: 323]) 2 mM extracellular Ca^2+^ was added and cells analyzed by flow cytometry (BD FACSAria Cell Sorter; BD Biosciences) at an excitation wavelength of 488 nm and an emission wavelength of 525 nm. The fluorescence intensity of ~1×10^6^ labeled cells was collected for each analysis, and the data are expressed as the median fluorescence intensity in arbitrary units from the average of at least three separate experiments. Data were collected using logarithmic amplification on 1×10^6^ cells, excluding cell debris.

### Quantification of gene expression (pro- and anti-oxidant genes and calcium regulators)

#### RNA extraction

Total RNA was extracted using Tri-Reagent (Invitrogen) extraction, chloroform phase separation, and isopropanol precipitation. Complimentary DNA (cDNA) was generated by reverse transcription (Sigma Aldrich) of 0.5µg of DNase treated total RNA using the random primer method (Invitrogen).

#### Real-time RT–PCR

Transcript levels for all genes unless otherwise stated (see below) were determined by real-time Taqman PCR using primer and probe sets formatted as pre-developed assays and run on 384 well low-density array plates (Applied Biosystems, Warrington, UK). Probes were labeled with 5′-FAM (carboxyfluorescein) and with 3′-10 TAMRA (Tetramethylrhodamine) as quencher. PCR was performed on a PerkinElmer 7700 cycler (Perkin Elmer Ltd., Dublin, Ireland) using the following steps 1) 2 min at 50 °C; step 2,) 10 min at 95 °C; step 3) 15 s at 95 °C; step 4) 1 min at 60 °C; step 5) repeat from step 3 for an additional 39 times.

Amplification of *NCX-1*, *PMCA-1*, and *PMCA-4* following hydrogen peroxide treatment of NLC cells was performed using the following primer sets; *NCX-1*, 5′-CTG GAA TTC GAG CTC TCC AC-3′, 5′-GGC ATC ATG GAG GTG AAA GT-3′; *PMCA1*, 5′-TTT CCA AAC ACT GCT TCT CTT C-3′, 5′-GGT CCA CAG ATG CAT TAC GA-3′; *PMCA4*, 5′-GAG CTT CCT GGA TAC CGA TG-3′, 5′-CTA GCT TGG TTG CCA CAC TG-3′. 18s rRNA was used to normalize threshold cycle number values (cT’s) in all PCR reactions. When using low-density array plates the NLC cells replicate for each gene were examined to establish if a reliable group mean could be obtained. When the groups mean lay within 1.5 cycles (2.5 fold difference) of the range of individual NLC cells replicates, the group mean was considered reliable. 2^-DcT was used to derive a fold difference in GLC cells compared to NLC cells. When original cT values for the target gene were above 40 cycles, this was designated as no detectable expression. Where no reliable average could be obtained for the NLC cells calibrator, comparative analysis with GLC cells was not pursued.

### SDS–PAGE and western immunoblot analysis

Total protein from confluent LC cells was obtained by lysing in RIPA buffer (50 mM Tris-HCl [pH 7.4], 1% NP-40, 0.25% sodium deoxycholate, 150 mM NaCl, 1 mM EGTA, 1 mM sodium orthovanadate, and 1 mM sodium fluoride) containing antipain (1 μg/ml), aprotinin (1 μg/ml), chymostatin (1 μg/ml), leupeptin (0.1 μg/ml), pepstatin (1 μg/ml), and phenylmethylsulfonyl fluoride (PMSF; 0.1 mM) and a phosphatase inhibitor cocktail (Sigma). Total protein (20 μg) was separated by SDS–PAGE followed by transfer to polyvinyl difluoride membrane (PVDF; 100 mV, 2 h; Millipore, Cork, Ireland). Following a 1 h blockade of membranes in 5% BSA (or 5% non-fat dry milk) in 0.1% Tween-20 tris buffered saline (TTBS; pH 7.4) membranes were incubated overnight at 4 °C with primary antibodies recognizing PMCA, NCX 1, sarco/endoplasmic reticulum Ca2+ ATPase (SERCA)2, and SERCA3 (all Santa Cruz Biotechnology, Santa Cruz, CA), and α-actin (Cell Signalling, Danvers, MA). Antibodies were incubated at 1:1,000 in 1% BSA/TTBS (Cell Signaling) or 1:150 in 5% non-fat dry milk/TTBS (Santa Cruz Biotechnology) overnight. Membranes were then washed in TTBS and incubated with 1:10,000 dilutions of HRP-linked secondary antibody (anti-mouse IgG, anti-rabbit IgG, and anti-goat IgG; Cell Signaling) and developed using enhanced chemiluminescence (ECL) reagents (Amersham Biosciences, Buckinghamshire, UK).

### Statistical analysis

Data are presented as mean±SD. All data were analyzed using unpaired *t*-test for analysis between two groups. Statistical significance was set at p<0.05 and denoted as * unless otherwise stated.

## Results

### Elevated intracellular ROS production, reduced anti-oxidant potential, and decreased mitochondrial membrane potential prevail in lamina cribrosa cells from glaucomatous donors

**Figure 1:**
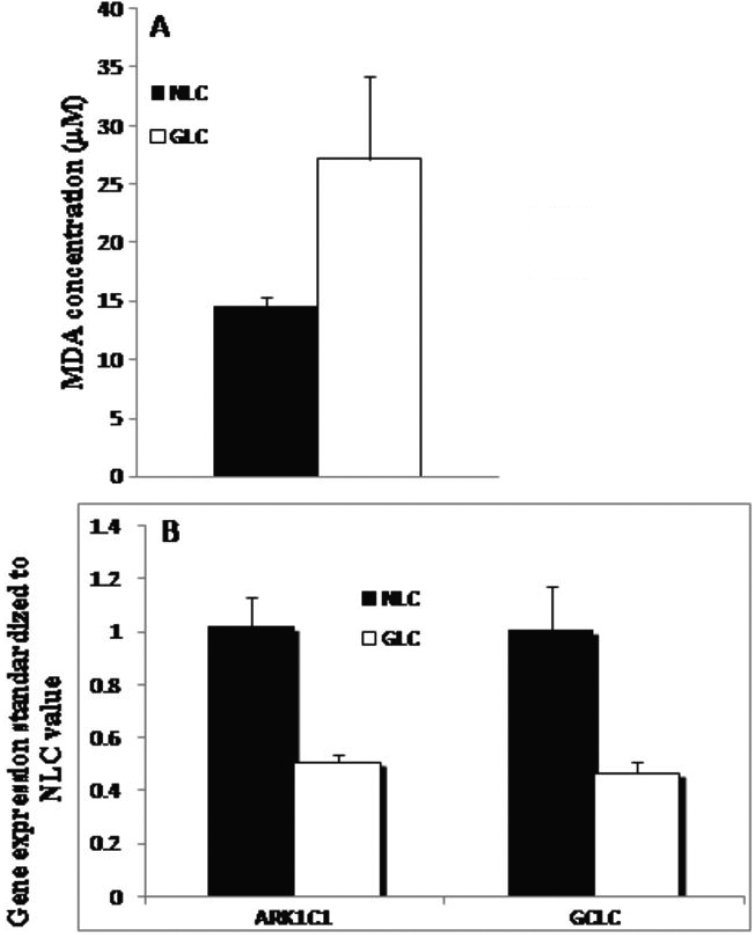
Increased ROS production and diminished anti-oxidant potential in GLC cells. **A**: Illustration of the levels of malondialdehyde (MDA), a naturally occurring product of lipid peroxidation (an indicator of oxidative stress) in LC cells. Increased expression of MDA was found in glaucomatous LC cells (GLC) when compared with LC cells derived from normal donors (NLC; 27.19±7.05 µM MDA versu 14.59 µM MDA±0.82, p≤0.05, n=3). **B**: mRNA expression of enzymes associated with maintenance of oxidative homeostasis was analyzed. As can be seen, expression of *AKR1C1* and *GCLC* are significantly lower in GLC cells (p*=*0.02) compared to NLC cells, expression normalized to NLC gene expression value. These results are suggestive of increased levels of oxidative stress and/or a compromised anti-oxidant state in cells from glaucomatous donors. Results are representative of 3 independent experiments with n=3 for NLC and GLC in each experiment.

We have previously shown that the mechano-sensitive pathway associated with cell membrane stretch in the normal LC cell is mediated through Ca^2+^ signaling [26]. Elevated Ca^2+^ can lead to mitochondrial depolarization and an increased generation of ROS. Levels of ROS production in NLC and GLC cells were determined using a TBARS assay (see Methods). GLC cells showed significantly higher ROS production compared with NLC cells (27.19±7.05 µM MDA versus 14.59 µM MDA±0.82, p<0.002, n=3; Figure 1A). In addition to measurement of ROS production we also examined the mRNA expression of enzymes associated with maintenance of oxidative homeostasis (Figure 1B). Hernandez et al. [37,38] had previously noted an altered expression of antioxidant enzymes, glutamate cysteine ligase catalytic subunit (GCLC) and aldoketo reductase 1C family member 1 (AKR1C1) in glaucomatous astrocytes of the ONH. AKR1C1 helps cells to reduce HNE (4-hydroxynonenal, a by-product of lipid peroxidation) into the non-toxic 1,4-dihydroxy-2-nonene (DHN) and GCLC, the first rate-limiting enzyme in the synthesis of glutathione. Expression of the above enzymes was significantly lower (p=0.02) in GLC cells compared with NLC cells (Figure 1B) suggesting a compromised anti-oxidant state in these glaucomatous cells.

**Figure 2:**
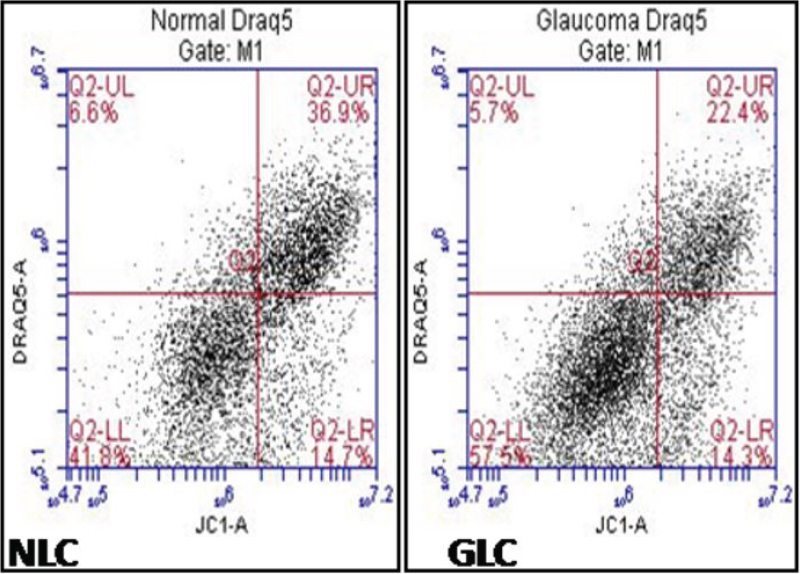
Decreased mitochondrial membrane potential (MMP) in GLC cells. JC-1 exists as a green-fluorescent monomer at low membrane potential, while at higher membrane potential, JC-1 forms red-fluorescent aggregates and it can therefore be used as a sensitive measure of changes in mitochondrial membrane potential. There is a substantial shift in fluorescence emission, indicating a decrease in MMP in GLC (57.5±6.8%) cells compared to NLC (41.8±5.3%). Results are representative of 3 independent experiments with n=3 for NLC and GLC in each experiment.

We subsequently evaluated mitochondrial membrane potential (MMP) in LC cells from both normal and glaucomatous donors (Figure 2) by using the fluorescent probe JC-1 and flow cytometry. JC-1 exists as a green-fluorescent monomer at low concentrations or at low membrane potential. However, at higher concentrations or higher potentials, JC-1 forms red-fluorescent aggregates and can therefore be used as a sensitive measure of mitochondrial membrane potential (ΔΨ_m_). As is evident from Figure 2 there is a substantial shift in the fluorescence emission [GLC (57.5±6.8%) cells compared to NLC (41.8±5.3%)], indicating a decrease of ΔΨ_m_ in LC cells obtained from the ONH region of donors with glaucoma implicating mitochondrial dysfunction in pathogenic damage to the ONH.

### Basal cytosolic [Ca^2+^]_i_ is elevated in glaucomatous LC cells

While ROS frequently function as cellular second messengers, at elevated levels they can be extremely damaging and disturb normal cellular physiologic function. We measured the [Ca^2+^]_i_ in NLC and GLC using flow cytometry after the cells were preloaded with the intracellular free [Ca^2+^]_i_ indicator fluo-4/AM in NLC and GLC cells. Addition of 2 mM extracellular Ca^2+^ elevates [Ca^2+^]_i_ in control LC cells and the increase in cytosolic Ca^2+^ (fluo-4/AM fluorescence) is more intense and has greater variability in GLC cells compared with NLC cells (p<0.001).

### Altered calcium transporters expression in lamina cribrosa cells isolated from glaucoma donors

**Figure 3:**
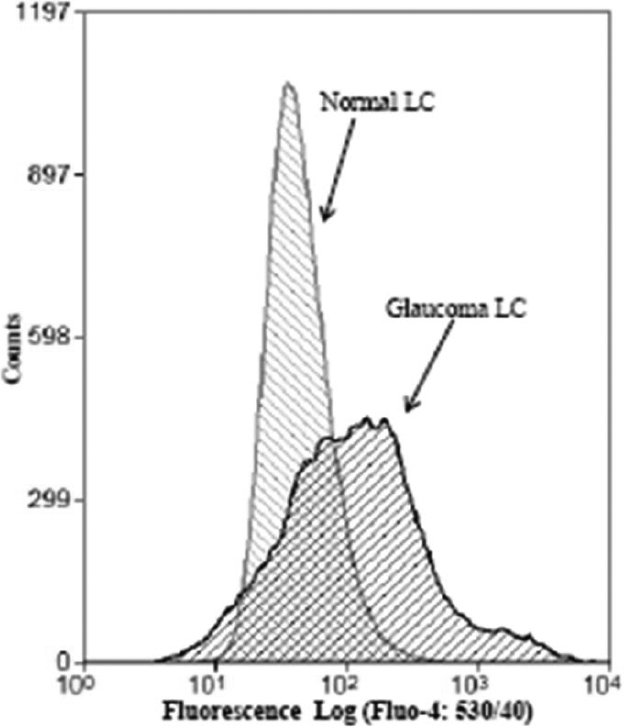
GLC cells show elevated cytosolic Ca^2+^. Flow cytometry was performed in Fluo-4/AM loaded NLC and GLC cultures. LC cells were contained in 1 ml isotonic Ca^2+^ free solution and the fluorescence intensity was measured. Addition of 2 mM extracellular Ca^2+^ induced a rise of cytosolic Ca^2+^ in both NLC and GLC and this response was is more intense and has greater variability in the GLC cells than in normal control LC cells. Results shown are tracings of a typical experiment with similar results observed in 3 separate experiments.

**Figure 4:**
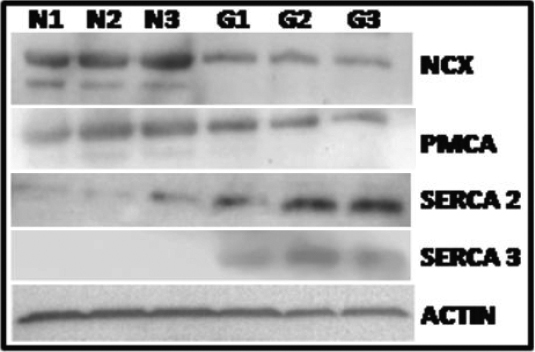
Expression of Ca^2+^ extrusion and storage systems are altered in GLC cells. Western blot analyses of Ca^2+^ extrusion and storage systems in NLC cells (n=3 cell lines) and GLC cells (n=3 cell lines). Protein lysates were examined for differential expression of the calcium extrusion components, PMCA, NCX and the SERCA storage pump. Overall, PMCA-1 and PMCA-4 expression is down regulated in GLC cells when compared to NLC cells, a similar pattern is also observed for NCX-1 and NCX-3. Expression of SERCA2 and SERCA3 is significantly upregulated in GLC cells compared to NLC cells. n=3 for NLC and GLC cells. Actin is used as a loading control for this experiment.

Based on our observation that intracellular Ca^2+^ levels are elevated in GLC cells (Figure 3) we determined whether there was differential gene expression of Ca^2+^ exchangers/pumps involved in the maintenance of Ca^2+^ homeostasis in GLC compared with NLC cells (Table 1) and confirmed the results at the protein level (Figure 4).

**Table 1:** Analysis of altered expression of Ca^2+^ transport system genes in NLC and GLC cells.

**Gene name**	**Mean CT value (±SD) NLC**	**Mean CT value (±SD) GLC**	**Fold difference in GLC**
*PMCA1*	15.4 (±0.4)	16.1 (±0.4)	**-0.4 p=0.02**
*PMCA2*	NRM in NLC calibrator		
*PMCA3*	NRM in NLC calibrator		
*PMCA4*	13.6 (±0.7)	13.6 (±0.2)	NSD
*NCX1*	NRM in NLC calibrator		
*NCX2*	NRM in NLC calibrator		
*NCX3*	NRM in NLC calibrator		
*SERCA1*	NRM in NLC calibrator		
*SERCA2*	20.1 (±1.3)	19.5 (±1.7)	NSD
*SERCA3*	19.1 (±0.2)	16.5 (±0.5)	**+5.87 p=0.01**

Real time PCR analysis of genes involved in the removal of cytosolic Ca^2+^ was performed; plasma membrane Ca^2+^ ATPase (*PMCA*) genes 1 to 4, Na^+^/Ca^2+^ exchanger (*NCX*) 2 and 3 and sarcoplasmic endoplasmic reticulum ATPase (*SERCA*) 1-3. The result of a comparative analysis of the expression of each of these genes is presented here as mean CT value (±SD) for both NLC and GLC. Gene expression is normalized to 18s. As is evident from the above table we observe a down-regulation in *PMCA1* expression in GLC compared to NLC and an up-regulation of *SERCA3* expression in GLC compared to NLC. NRM indicates that there was no reliable mean obtained for NLC values possibly due to low expressional levels. NSD indicates that there was no significant difference between the mean CT value (±SD) for both NLC and GLC.

#### Plasma membrane Ca^2+^-ATPase (PMCA)

A 40% decrease in PMCA1 mRNA expression (−0.4±0.1 fold p<0.05) was observed in GLC compared to NLC cells. No significant differences were observed in the mRNA expression of either the *PMCA3* or *PMCA4* genes, while no appreciable expression of *PMCA2* was detected (Table 1). Western blotting using a pan anti-PMCA antibody demonstrated reduced PMCA expression at the protein level in GLC cells (Figure 4).

#### Na^+^/Ca^2+^ exchanger (NCX)

Examination of mRNA levels of the three *NCX* genes (*NCX1*, *NCX2*, and *NCX3*) failed to show a steady pattern of expression in NLC, thereby preventing reliable comparison of expression rates between NLC and GLC (Table 1). However, western blotting using a pan anti-NCX antibody demonstrated down regulation of NCX protein in GLC compared to NLC cells (Figure 4).

#### Sarco/endoplasmic reticulum Ca^2+-^ATPase (SERCA)

A significant upregulation of *SERCA3* mRNA was observed in GLC compared to NLC cultures (5.87±1.83 fold, p<0.05, Table 1) and was confirmed at the protein level using a SERCA3 specific antibody (Figure 4). The mRNA levels of *SERCA1* and *SERCA2* were not significantly different between NLC and GLC, although an apparent increase in SERCA2 protein was observed by western immunoblotting. No significant differences were observed in the mRNA expression of the Golgi apparatus associated Ca^2+^ ATPase 2C1 pump between NLC and GLC cells.

### Subjection of normal LC cells to oxidative stress (hydrogen peroxide) results in the down-regulation of key Ca^2+^ extrusion transporters (NCX-1, PMCA-1 and PMCA-4)

**Figure 5:**
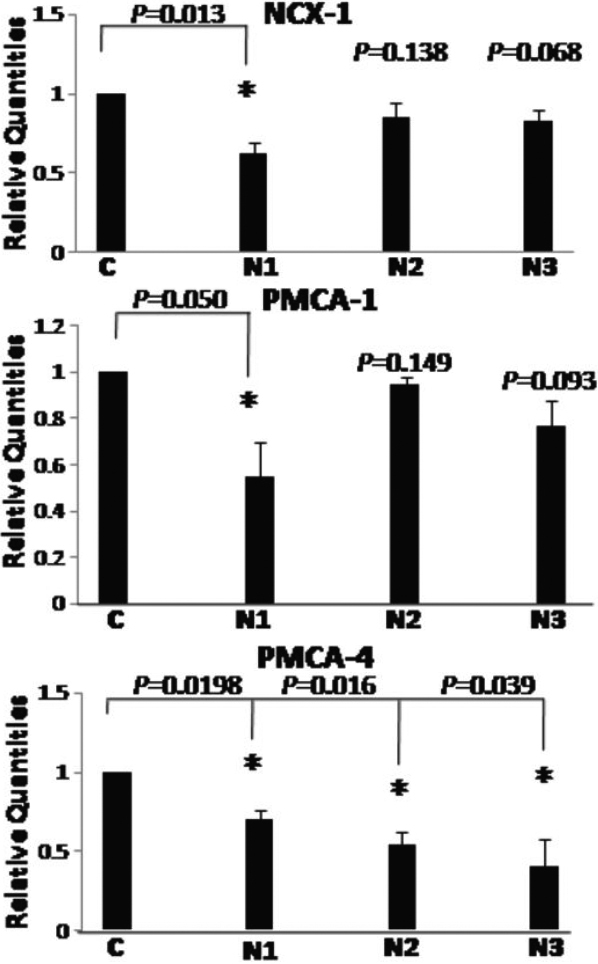
Hydrogen peroxide treatment of NLC cells causes a down-regulation in expression of Ca^2+^ extrusion system (*NCX-1*, *PMCA-1*, and *PMCA-4*) expression. NLC cells obtained from 3 donors (N1, N2, and N3) were treated with 200 µM H_2_0_2_ for 1 h, RNA isolated and gene expression of *NCX-1*, *PMCA-1*, and *PMCA-4* was analyzed by quantitative PCR. Control (C) NLC cells were included for expressional comparison and assigned the arbitrary value of 1. Introduction of H_2_O_2_ significantly reduced expression of Ca^2+^ extrusions systems (*NCX-1*, *PMCA-1*, and *PMCA-4*) in some cell lines. Although, not all of the reductions are statistically significant; most approach significance and exhibit a downward trend. Results are representative of 3 individual experiments and significant results are denoted by * (p<0.05).

Previous studies have shown that oxidative stress (hydrogen peroxide) can alter the expression of Ca^2+^ channels [34,37,39] thus, we investigated the redox modulation of Ca^2+^ transport proteins in NLC cells following exposure to H_2_0_2_ (Figure 5). Cells were treated with 200 µM H_2_0_2_ for 1 h before RNA isolation and assessment of Ca^2+^ extrusion transporter expression by RT–PCR. Results demonstrate that NLC cells subjected to H_2_0_2_-induced oxidative stress results in a down-regulation of *NCX-1*, *PMCA-1*, and *PMCA-4*. While some donors show a non-statistical decrease, all approached significance.

## Discussion

ROS normally exist in a physiologic harmony with cellular antioxidants. Oxidative stress occurs when this critical balance is disrupted or compromised. In this study we demonstrate increased ROS production (Figure 1A), compromised anti-oxidant capacity (Figure 1B), mitochondrial dysfunction (Figure 2) and dysfunctional Ca^2+^ homeostasis (Figure 3 and Figure 4, Table 1) in GLC cells compared to NLC cells. ROS generation was found to be present at significantly higher levels in the LC cells obtained from glaucomatous donors (Figure 1A) compared to normal LC cells. Importantly, the capacity of the GLC cell to counteract this increase in ROS production also appears to be impaired because expression levels of anti-oxidant enzymes (AKR1C1 and GCLC; Figure 1B) are diminished in GLC cells (p=0.02). Hernandez et al. [37,38] also showed altered expression of these enzymes in glaucomatous astrocytes of the ONH. There is previous evidence of mitochondrial dysfunction in glaucoma and mitochondrial dysfunction participates in RGC death [21,22,40-43]. We found significantly decreased MMP in GLC compared to NLC cells (Figure 2) indicating impairment of normal mitochondrial function in the disease state. Of particular significance in the context of this study is the fact that mitochondria play a key role in switching normal Ca^2+^ signaling to signals for cell death signaling during oxidative stress conditions [13]. Furthermore, it has been postulated that ROS can impair mitochondrial respiration and depolarize the mitochondrial membrane thereby decreasing the organelles ability to buffer Ca^2+^ [43-45].

Our data are consistent with previous studies that show increased levels of oxidative damage markers including peroxidised lipids, oxidative DNA damage and reduced antioxidant potential in various ocular tissues of glaucoma patients. This supports oxidative damage as a characteristic of this disease. Pro-oxidants have previously been described in the aqueous humor of glaucoma patients [46-48] and anti-oxidants such as superoxide dismutase (SOD) are expressed in TM cells [49]. Therefore total reactive antioxidant potential may be useful as an oxidative stress marker in the glaucoma patient. Oxidants cause a rapid increase in intracellular Ca^2+^ concentrations in diseased cells [50-52] and can cause a release of Ca^2+^ from internal stores [53-56]. The effects of oxidants on Ca^2+^ signaling can vary from stimulatory to suppressive/inhibitory depending on the type of oxidant, concentrations and duration of exposure. In this study we have gone some way to describing the effects of oxidative stress (H_2_0_2_) on calcium transporter expression (Figure 5), illustrating oxidant (H_2_0_2_) induced down-regulation of Ca^2+^ extrusion systems (*NCX-1*, *PMCA-1*, and *PMCA-4*) in NLC cells. This decrease in expression mimics the endogenous decrease in expression of these channels found in GLC compared with NLC cells (Table 1) which may in part be responsible for elevated [Ca^2+^]_i_ levels noted in the GLC cells. These findings are in agreement with numerous studies which have illustrated redox modulation of Ca^2+^ transporter systems [34,35,39,57]. While oxidant regulation of Ca^2+^ transporter systems is unlikely to be the sole mechanism accountable for Ca^2+^ dysregulation in the GLC cell these initial findings provide a basis for further investigation.

To maintain Ca^2+^ fluctuations within physiologic levels, cells have developed very efficient regulatory systems. [Ca^2+^]_i_ is determined by the combined action of buffers, pumps and exchangers [24]. Our findings of elevated [Ca^2+^]_i_ levels in GLC cells compliment recent work by Niittykoski et al. [58] and Sappington et al. [59] who independently demonstrated a similar increased basal Ca^2+^ level in glaucomatous RGC cells.

Our data illustrates altered basal Ca^2+^ levels in GLC cells and describe changes in the transcriptional and translational alterations of complexes involved in the extrusion and reuptake channels for Ca^2+^ in GLC cells. PMCA’s are transport proteins in the plasma membrane of cells that serves to remove Ca^2+^ from the cell [60]. A 40% decrease in PMCA1 expression in GLC cells was observed (Table 1) may account for the increase in [Ca^2+^]_i_ we observe in GLC cells (Figure 3). Protein analysis identified a reduction in both NCX1 and NCX3. NCX exchangers can move calcium into (forward mode) and out (reverse mode) of the cell depending on the net electrochemical driving force [61]. This exchanger has a low affinity for Ca^2+^ ions but a relatively high extrusion rate. This makes the NCX exchanger important in regulating local spikes in Ca^2+^ used to signal in rapid responses to an event, like rapid increases in cell membrane stretch.

SERCA resides in the sarcoplasmic and endoplasmic reticulum (SR). It is a Ca^2+^ ATPase that transfers Ca^2+^ from the cytosol of the cell to the lumen of the SR/ER at the expense of ATP. SERCA pumps perform the crucial function of refilling the depleted Ca^2+^ stores and therefore constitute an integral part of the cellular Ca^2+^ homeostasis circuitry. Here we have shown higher levels of SERCA2 and SERCA3 proteins in GLC cells. Interestingly, an increase in SERCA2 expression was also observed after induction of the endoplasmic reticulum stress response in the neuroendocrine cell line, PC12 [62] and SERCA pump dysfunction has been implicated in several diseases [63]. This increase in SERCA2 and SERCA3 is consistent with our findings of increased basal calcium levels in GLC cells (Figure 3). Increasing intracellular Ca^2+^ has been shown to upregulate *SERCA3* mRNA expression in other tissues [64]. SERCA3 is also known to be more resistant to oxidative stress than the other SERCA pumps [65]. Therefore, SERCAs maybe preferentially expressed in tissues exposed to chronically elevated levels of oxidative stress, such as in the hypoxic microenvironment in the LC region of the glaucomatous ONH.

In summary, we have clearly shown that conditions of oxidative stress prevail in GLC cells. These cells also exhibit impaired mitochondrial function and marked abnormalities of Ca^2+^ homeostasis which correlate with elevated cytosolic Ca^2+^. Whether anomalous Ca^2+^ homeostasis in GLC cells is a primary pathological event or a consequence of other pathological changes has yet to be elucidated. Future anti-glaucoma therapies may target oxidative stress and Ca^2+^ overload in the LC cell.
